# Governing industry involvement in the non-communicable disease response in Kenya

**DOI:** 10.1186/s12992-021-00776-3

**Published:** 2021-10-20

**Authors:** Tobias Bünder, Catherine Karekezi, Veronika Wirtz

**Affiliations:** 1 Hertie School, Friedrichstraße 180, Berlin, 10117 Germany; 2 Non-communicable Diseases Alliance Kenya (NCDAK), KMA Plaza Apartments, Block C Unit 5.2, Mara Road – Upper Hill, P. O Box 5337, Nairobi, Kenya; 3 Kenya Diabetes Management & Information Centre, Jadala Place, 2nd Floor Office 2.1, Ngong Lane, Ngong Road, P. O. Box 45099 -, Nairobi, 00100 Kenya; 4 Department of Global Health, Boston University School of Public Health, 801 Massachusetts Avenue, Boston, MA 02118 USA

**Keywords:** Governance, Kenya, Pharmaceutical industry, Non-communicable diseases, Public-private partnerships, CSR

## Abstract

**Background:**

In low- and middle-income countries (LMICs), multinational companies have become increasingly involved in addressing public health challenges. Dealing with companies as partners in health sector development creates new challenges for governments. We sought to develop an approach to assess the existence and effectiveness of governance structures that can ensure that industry-led public health initiatives contribute to development.

**Methods:**

We developed a governance assessment tool based on the principles of the Paris Declaration for Aid Effectiveness and other related agreements. We applied it to the case of pharmaceutical companies’ involvement in the Kenyan response to non-communicable diseases (NCDs). We gathered data for analysis through 46 stakeholder interviews and reviewing documents.

**Results:**

The Kenyan government has informal norms in place regarding program governance and strategy, but it has yet to issue formal regulations. While enabling elements exist that support initiatives to develop in alignment with these norms, implementation is often hindered by a lack of resources. Currently, broad stakeholder support for filling these gaps has created a window of opportunity for action.

**Conclusion:**

The application of the proposed assessment tool illustrates its viability for assisting companies and governments alike in defining governance needs for industry-led public health initiatives. Our findings in Kenya provide example considerations for LMICs working to integrate industry-led public health programs into the health system. Bilateral and multilateral donors also have important roles in strengthening LMICs’ capacities to govern multinational corporations’ contributions to NCDs in particular, and development in general.

## Background

Transnational corporations, including those in the pharmaceutical industry, have gradually been taking on more proactive roles in national-level development, acting as development partners with governments and non-governmental organizations (NGOs) in many low- and middle-income countries (LMICs) [1–3]. Especially the passing of the UN Agenda 2030 has again emphasized the ambition of countries and international organizations to work with and through the private sector to achieve the Sustainable Development Goals [4–6]. Both practitioners and scholars suggest that companies can bring different capacities and additional resources to the table to solve problems that require multi-sectoral approaches [2, 7, 8].

In global health, the number of access-to-medicines and other global health initiatives involving pharmaceutical companies or their foundations has significantly increased in recent years [9]. Pharmaceutical companies have taken on especially strong roles as development partners for LMICs addressing non-communicable diseases (NCDs) [10]. The industry has both expertise and strategic interests in supporting LMIC’s NCD response, as medicines for NCDs constitute the core business of many pharmaceutical companies [11]. Most other global health donors are still shying away from NCDs and continue to focus on other health challenges [12].

Research on the role of business in development has repeatedly pointed out that there is – at best - only anecdotal evidence to support the assumed positive effect of multinational companies acting as development agents [13–16]. Thus, global health scholars and policymakers are divided over the desirability of the growing involvement of pharmaceutical and other companies in public health efforts in LMICs. Some perceive it as an opportunity to tap into additional resources and expertise [17, 18]. Those with more critical perspectives are concerned that companies use their involvement to gain more influence in global health governance [19, 20]. Critics have also identified risks of industry involvement in countries’ health systems [21, 22]: because corporate interests do not necessarily align with public health priorities, corporate development initiatives may lead to undesirable diversion of limited local resources. Corporations might also gain undue influence through these activities that they could later use to steer future decision-making to their own advantage. To address these concerns, advocates have suggested that it is important for LMICs’ governments to actively steer and regulate private sector involvement [17, 21, 22]. The World Health Organization (WHO) has developed a checklist for governments dealing with corporate access to medicines initiatives [21]—however, gaps remain in understanding the extent to which this or other proposed structures and processes for stronger governance have been adopted by countries.

Thus, this paper asks: how and to what extent can LMIC governments govern industry involvement in development initiatives? What challenges do governments face, and what lessons can we derive from their current efforts? The paper begins by reviewing the principles for governance of health and development initiatives that we integrated into an assessment tool. We then use the tool to focus on the case of Kenya, which has been a leader among LMICs in experimenting with industry involvement in responding to the expanding challenge of NCDs.

Thereby the paper aims to assess the degree to which the Kenyan government has already implemented governance structures and processes to promote the effectiveness of pharmaceutical corporations’ involvement in the NCD response. It highlights some of the challenges this effort has faced and derives lessons from Kenya’s experience for other countries dealing with a growing number of industry-led programs.

### The Paris declaration on aid effectiveness as a governance framework

In 2005, more than 100 donor and aid-receiving countries, as well as major international NGOs and multilateral institutions, agreed on the Paris Declaration on Aid Effectiveness [23], which lays out a set of five partnership principles for development: country *ownership* of development efforts*, alignment* with local systems and priorities, *harmonization* among initiatives, *managing for results*, and mutual *accountability*. The 2008 Accra Agenda for Action [23] affirmed these principles and added *stakeholder inclusivity* as a sixth element. While the effectiveness of donor aid also depends on other factors, many of which are case- or country-specific, these principles represent a set of necessary or enabling conditions and rights-based norms.

The Paris Declaration is the leading framework on how to conduct development cooperation. The Paris Declaration does not explicitly address the private sector, but there is no reason to assume that its principles would be less valid for corporations when they act as development partners [24]. International global health donors further committed to a sector-specific application of these principles in the 2007 International Health Partnership [25]. In 2016, IHP evolved into the UHC2030 coalition, whose “Global Compact” also directly refers to the Paris Declaration [26].

There is an established practice of referring to these principles in academic work that analyzes and evaluates corporate global health efforts [20, 22, 27–29]. Recent actions to lay out guidelines for the pharmaceutical industry’s global health efforts, such as the WHO policy brief or the partnership principles of the industry’s Access Accelerated alliance, also build on the Paris Declaration [21, 30]. Table 1 shows how the guiding principles are presented in these different documents, from the general aid effectiveness agenda to the specific case of NCDs. The column on the far-right offers several examples of why following aid principles is not necessarily in companies’ interests. These include concerns about the costs involved in collaboration processes, losing control of corporate resources, and being forced to act against their own profit-making interests. Given potential inconveniences and conflicts of interest, governments cannot rely on companies’ adherence to established, but voluntary, principles in international aid.

**Table 1:** Aid effectiveness principles and potential challenges

Principle		Universal	Health, in general	Health, industry initiatives		Potential challenges for industry as development partner
		Paris Declaration & Accra Agenda for Action (2005/2008) [23]	UHC 2030 Global Compact (2018) [26]	WHO Policy Brief for governments (2017) [21]	Access Accelerated guiding principles for industry (2019) [30]	
**Program strategy**	***Alignment***	“Donors base their overall support on partner countries’ national development strategies, institutions and procedures”	“All partners should ensure their efforts are evidence-based and align with national priorities and policies”	“Ensuring that initiatives abide by all national regulations; align with national health plans and other development plans and goals”	“Align with government priorities and support national efforts to build sustainable access to NCD prevention, treatment and care services”	• Corporate strategic interests might not align with country priorities. • Preference for creating parallel company-controlled structures, over investing in adapting and improving existing systems.
	***Harmonization***	“Donors’ actions are more harmonized, transparent and collectively effective.”	“Ensure coordination and alignment of health system strengthening efforts at global, regional and country levels and appropriate linkages with other sectors.”	“Harmonization and coordination with existing programs and future initiatives should also take place to avoid duplication”	“Build a collaborative network of member companies, partner organizations, and other key stakeholders to share knowledge and support a more coordinated collective response to NCDs”	• Competition among companies for reputation and influence may impede willingness or ability to coordinate. • Harmonization can be more costly and slower than independent action.
	***Ownership,*** ***stakeholder inclusion***	“Donors commit to respect partner country leadership and help strengthen their capacity to exercise it.” “All partners - including donors,foundations and civil society - participate fully”	“Making health systems everybody’s business – with engagement of citizens, communities, civil society and private sector”	“Decision-making should be open to the public and include NGOs and other non-governmental stakeholders.”	“Foster collaboration and open communication with local stakeholders at all stages of program development, execution and evaluation”	• Thorough stakeholder involvement requires additional time, investment, and complexity. • Giving away control and influence can jeopardize any preconceived ideas and priorities for engagement.
**Program implementation**	***Managing by results***	“Developing countries and donors shift focus to development results and results get measured.”	“Accountability for results”	“Process for monitoring and evaluation has been established”	“Apply appropriate monitoring and evaluation processes to understand how a program is contributing to its stated goal(s), including improved health, and broadly share learnings from successes and challenges”	• Substantial investment of financial and management resources that many corporations are not willing to make.
	***Accountability***	“Enhance mutual accountability and transparency in the use of development resources”	“All partners should … recognise their accountability to people and communities.”	“Have strong mechanisms to ensure financial, performance, and public accountability”	“Establish accountability measures, manage expectations, and build mutual understanding”	• Fear the reputational effects of reporting negative results. • Fear of sharing information considered proprietary.

### Pharmaceutical industry-led NCD initiatives in Kenya

With 27 individual corporate programs by 11 different pharmaceutical companies running in January 2020, Kenya was the country with the greatest number of industry-led NCD programs, according to the monitoring platform Access Observatory (www.accessobservatory.org). Industry-led programs have explicit social goals and are often structured as cross-sector partnerships between companies, NGOs, and government agencies [3]. Yet, they differ from more traditional global health partnerships [31] in that they are “designed and co-financed by companies and companies take responsibility and credit for them” [28]. Whilst most companies work through their Kenyan business or their in-house corporate responsibility department, two of the eleven companies primarily work through their corporate foundations. Even though foundations are technically independent, in this paper, we treat them as extensions of their mother companies, as companies take credit for their work and corporate executives usually lead the foundations’ governance. In Kenya, industry-led programs were mainly focused on cancer care, diabetes, and cardiovascular diseases. While some programs were primarily providing access to health products, the majority also involved elements of strengthening or providing health services, such as supporting health worker training, sponsoring screening campaigns and even development of policy and guidelines [32].

The international pharmaceutical industry has always had a large footprint in Kenya. Many companies have established their regional offices in the country, which constitutes one of Africa’s most promising growth markets, works as a regional logistics hub, and has a market-friendly political system. The launch of Access Accelerated (AA) in January 2017 reinforced industry involvement in Kenya’s NCD response. AA is an industry-led alliance of more than twenty pharmaceutical companies collaborating with the World Bank, PATH, NCD Alliance, City Cancer Challenge, World Heart Federation, and RTI International to improve NCD care. AA engages in its own projects as well as serving as a coordination and standard-setting mechanism for industry-led NCD initiatives. In 2018, AA selected Kenya to be one of two pilot countries for deeper industry involvement in the NCD response.

Similar to global trends, NCDs have recently increased in political relevance in Kenya. The first NCD policy to be developed in Kenya was the National Diabetes Strategy in 2010, supported by the World Diabetes Foundation (WDF), which is funded by the company Novo Nordisk [33]. With increasing NCD prevalence and disease burden, more civil society organizations began pushing for NCDs, especially cancer, to be taken seriously [34]. As a result of this attention and ongoing advocacy, various national NCD policies and legislative documents have been developed [35–38]. However, implementation of the policies has been inconsistent. It differs by county, as Kenya’s devolved constitution grants fiscal, political and operational responsibility for health services to county-level governments [39]. Further, there is a persistent lack of available resources for NCD services. Only a few development partners have thus far engaged in the NCD response, and domestic funding for health remains too limited to cover the necessary budgets. In the face of the growing public health need, and in light of the lack of available resources, national- and county-level actors turned to pharmaceutical companies as possible development partners in the Kenyan NCD response.

In line with the devolution process and the “Kenya Health Policy 2014-2030” [40], the Kenyan government has been working on improving governance structures and processes to manage health sector partnerships. Most notably, the Ministry of Health, with support from WHO, is currently developing a new “Kenya Health Sector Partnership and Coordination Framework” [41], which aims to guide management of all types of partnerships, including with NGOs and the private sector, as well as with bilateral and international donors. This remains an ongoing process, and thus far has not primarily focused on industry-led programs.

## Methods

### Data collection

To assess how Kenya has implemented governance structure and processes for industry-led NCD programs, this paper builds on data that were collected from primary and secondary sources in two phases. Between June and December 2019, we collected gray literature (including government documents, reports by international organizations or donors, academic articles) on the Kenyan response to NCDs; we also gathered reports from AA and the pharmaceutical companies about industry activities in the country. We captured existing stakeholder statements about industry-led NCD initiatives that we found in online news media and in videos of public events such as NCD stakeholder forums and panel discussions available on YouTube. We also extracted the Access Observatory’s data for Kenya to get an overview of which companies were working on which issues, and with which partners and strategies.

Based on the findings from that phase of data collection, we mapped stakeholders to identify organizations involved in the NCD space. Following a snowball sampling strategy, we started interviewing existing contacts within these organizations and asked them for links to other stakeholders. In total, we conducted 46 semi-structured interviews with stakeholders from five sectors: the public sector (national and county government agencies and ministries); the private sector (transnational and local pharmaceutical companies, industry associations); civil society (patient and disease advocacy organizations, NGOs); international organizations (donor agencies, UN agencies); and the health sector (medical professional associations, health service delivery institutions). All interviewees were either directly managing projects involving pharmaceutical companies or held senior management positions in their respective organizations. The interviewees’ organizations are listed in Table 2. The interviews, which were recorded, lasted between 26 and 103 min. Subsequently, we transcribed all interviews. We obtained the interviewees’ consent to record their interviews and assured them that they would remain anonymous. Thus, quotes are not attributed to names, job titles, or organizations, but only refer to the interviewee’s sector (government, health services, civil society, pharmaceutical industry). The project underwent ethics review by Strathmore University’s Institutional Review Board (SU-IERC0574/19).

**Table 2:** Overview of interviewees by sector

Sector	Organizations	Number
Public sector	Machakos County Ministry of Health, Meru County Ministry of Health, National Ministry of Health Department of NCDs, National Cancer Institute, Pharmacy and Poisons Board,	7
Civil society	Amref, Beyond Zero Campaign, Beth Mugo Cancer Foundation, Christian Health Association Kenya, Doctors Without Borders Kenya, Kenya Hospice and Palliative Care Association, Kenya Network of Cancer Organizations, Kenya Red Cross Society, NCD Alliance Kenya, Women for Cancer	14
Health sector	International Cancer Institute, Kenya Cardiac Society, Kenya Society for Hematology and Oncology, Meru Teaching and Referral Hospital, Nairobi Hospital	5
Private sector	Access Accelerated, Biodeal Laboratories, Kenya Association of Pharmaceutical Industries, Kenya Healthcare Federation, Merck, Medtronics, Novartis, Roche, Takeda,	15
International organizations	Development Partners in Health Kenya, GIZ, WHO, World Bank, UN SDG Partnership Platform	5
	Total number of interviews conducted:	46

### Data analysis

While different tools exist to assess the internal governance of individual programs and partnerships [27, 42], we did not find any framework to study the overarching governance of involving industry-led NCD programs in the health system. Thus, we developed an assessment tool based on the principles of the Paris Declaration. Our goal was to identify a set of governance elements that governments could put in place to shape how companies adhere to these principles. In this framework we grouped the principles of *alignment* and *harmonization* together, as both affect the design of program strategies. *Management by results* and *accountability* were analyzed together as benchmarks for governing program implementation. Finally, *ownership* and *stakeholder inclusion* were included as cross-cutting procedural principles, highlighting the roles that governments and affected groups should play in deciding about strategy as well as being involved in program implementation.

We identified both *regulatory* and *enabling* elements [43]. Regulatory elements are rules and norms about how corporations design and govern programs. These could be either formalized in policies or guidelines or just exist as informal norms. Regulatory elements have sanction mechanisms for non-compliance—these may be formal or informal, such as refusing to grant permission for program activities, withdrawing public resources, or damaging a company’s reputation. Enabling elements are any activities or structures that make it easier for corporations to adhere to the principles, such as provision of public data for needs assessments, or conducting stakeholder forums that corporations could use for better harmonization. In sum, country governance frameworks can shape the adherence of companies to the guiding principles in two ways: turning guiding principles into enforceable local rules and norms, and by assisting companies to set their programs up according to the principles.

Our selection of governance elements for assessment draws on three sources: First, on thoughts about governance frameworks for cross-sector partnerships [43, 44]. Second, we adopted suggestions made by the WHO for country responses to incorporate access to medicine initiatives which we described in the introduction and Table 1. Third, we identified relevant governance elements from the draft “Kenyan Health Sector Partnership and Coordination Framework” [41], which remained under review at the time of writing. We assigned each element to a single principle of the Paris Declaration for parsimony’s sake, even though some may be associated to multiple principles. Review meetings, for example, are certainly both important for *managing by results* and *accountability* alike.

Table 3 presents an overview of the assessment tool. The left column lists the Paris Declaration Partnership principles. The next column includes the regulatory and enabling elements as described above. The column furthest on the right lays out how each governance element can shape adherence to a given principle and as well as questions to assess a specific case. We used the assessment questions to code our interview transcripts with the help of the software MAXQDA and to review archival data in order to obtain answers to each individual question of our framework. If interviewees had conflicting perspectives on a question, we corroborated accounts through additional interviews or document review and report these nuances in the results section.

**Table 3:** Assessment tool

Partnership principles		Governance elements	Rationale for how element shapes adherence	Assessment questions
Program strategy	Cross-cutting	Regulation	Stating and implementing the government’s expectations about designing program strategy (e.g. outlining requirements for needs assessments or stakeholder involvement)	• Do formal policies or legislation exist that regulate program design? • If not, do clearly stated informal norms exist of what government expects from companies in this regard? • In how far are these rules or norms backed up with sanctions to enforce compliance?
	Alignment	Direct government support	Assisting companies in aligning with country priorities	• Does government support program design processes with public resources (staff time, funds etc.)?
		Provision of strategies and policies	Identifying a government strategy with which companies can align their programs	• Do sector strategies exist for companies to align with? • Is this information accessible for companies?
		Provision of data	Finding or generating data so companies can assess needs and align accordingly	• Does government provide data (e.g., on NCD prevalence and health system capacities) for needs assessments? • Is this information accessible for companies?
	Harmonization	Mechanisms for information sharing among partners	Sharing knowledge about stakeholders’ activities to enable harmonization	• Does a registry of active NCD programs exist for better harmonization? • Is it complete and updated regularly? • Can companies access this information? • Does government host an exchange structure for partners to plan jointly? • Is it open for companies?
	Ownership and stakeholder involvement	Structures for stakeholder engagement	Identifying and participating in existing engagement structures makes it easier for companies to broadly consult stakeholders	• Does the government host stakeholder engagement structures? • Do companies have access to them?
Program implementation	Cross-cutting	Regulation	Stating and implementing government expectations regarding the design of program governance (e.g., outlining requirements for representation on governance boards or M&E systems)	• Do formal policies or legislation exist that regulate program governance? • If not, do clearly stated informal norms exist of what government expects from companies in this regard? • In how far are these rules or norms backed up with sanctions to enforce compliance?
	Managing by results	Results framework	Guiding companies in setting up M&E systems	• Does the government provide a unified results framework that companies can build on?
	Accountability	Reporting structures	Offering platform for reporting results and creating transparency	• Does the government provide a public reporting framework where results can be shared transparently?
		Government oversight	Accountability through participation in governance structures of individual programs	• Does government join governance structures of corporate programs?
		Review meetings	Providing space for companies to broadly present and discuss results	• Does government host regular review meetings where companies can report on progress?

## Results

### Partnership principles in program strategy

Kenya’s National NCD Strategy 2015–2020 [35] underlines a need for cross-sector partnerships, but it does not specify expectations about how partnerships develop their strategies. Although there are no formal policies or laws guiding the design of industry-led NCD programs, informal local norms exist that govern how companies should act if they intend to launch a NCD program (see Table 4 below for a summary). In our interviews, representatives of the national government clearly stated their demands for strong government ownership, including expecting to be consulted on any corporate activities that are linked to the health system. One industry representative recognized this:

*“We strongly engaged with the NCD department in the Ministry of Health. Together we came up with a good approach. They were of the mindset of wanting to work with pharma in improving NCD care. Their only request was to not create parallel health systems and align well. If we kept them informed about anything we did, they would be happy to work alongside us.” (Interview #31, industry representative)*

As the quote alludes to, technical experts in the public sector explicitly demanded that companies developed programs that aligned with local priorities and systems. Since companies often rely on the public health infrastructure for the implementation of their programs or need to cooperate with regulatory bodies, the government has leverage opportunities to pressure the companies to meet its expectations.

**Table 4:** Kenya Results: Program strategy

Partnership principles		Governance elements	Assessment questions	Assessment of the situation in Kenya
**Program strategy**	Cross-cutting	Regulation	Do formal policies or legislation exist that regulate program design?	• No formal rules or laws existed at the time of the research. • The health sector partnership framework was under development.
			If not, do clearly stated informal norms exist of what government expects from companies in this regard?	• Government expects to be consulted on programs that interact with the health system. • Government and civil society expect companies to use NCD technical working groups for stakeholder consultation.
			In how far are these rules or norms backed up with sanctions to enforce compliance?	• Only informal sanctions are currently in place to pressure companies into meeting government expectations (e.g. through non-cooperation or withholding licenses).
	Alignment	Direct government support	Does government support program design processes with public resources (staff time, funds etc.)?	• MoH assigns technical teams to support program development. • MoH lacks sufficient capacity to do this for all industry-led programs.
		Provision of strategies and policies	Do sector strategies exist for companies to align with?	• A broad set of policies and strategies are in place at national level. • County development plans do not always exist and are often not costed.
			Is this information accessible for companies?	• Existing strategies are publicly accessible. • A complete and easily database is not available.
		Provision of data	Does government provide data (e.g. on NCD prevalence and health system capacities) for needs assessments?	• Publicly available data are not always complete or updated.
			Is this information accessible for companies?	• Health data are only partially accessible, but companies were able to work with KEMRI for better access to data in some cases.
	Harmonization	Mechanisms for information sharing among partners	Does a registry of existing NCD programs exist for better harmonization?	• The government conducted a mapping exercise in 2018 and results are available by request; beyond that, a government registry is not available. • Access Observatory (AO) exists as a privately funded alternative
			Is it complete and updated regularly?	• The 2018 mapping was not comprehensive and remained a one-off project. • AO is also not comprehensive. It is updated annually, but its future depends on AA’s continued funding.
			Can companies access this information?	• The AO is publicly accessible. • The 2018 mapping information was not published, but could be provided on request.
			Does government host an exchange structure for partners to plan jointly?	• There is no regular public structure. There was a private initiative: AA country team hosted two large-scale networking meetings. • The Development Partners in Health Roundtable is the leading coordination platform in the health sector where partners meet on a regular basis.
			Is it open for companies?	• AA/MoH meetings were company-focused. • Existing donors have thus far neglected NCDs and do not accept corporations as development partners. Thus, the Roundtable has yet to invite corporations to its meetings.
	Ownership and stakeholder involvement	Structures for stakeholder engagement	Does the government host stakeholder engagement structures?	• An NCD Interagency Coordinating Committee and different technical working groups (TWGs) on specific NCD themes include various stakeholders. • The TWG structure was only established effectively in 2019. Meetings are still irregular.
			Do companies have access to them?	• Companies can make use of these TWGs to discuss the design of their NCD programs, but are not full members.

However, the degree of government representatives’ insistence on its norms can vary on a case-by-case basis. According to other accounts, norms have at times been undermined. A civil society representative, for instance, asserted that high-level political leaders sometimes agree to companies’ preferences before technical experts were consulted.

*“Companies will not come and negotiate with the Ministry’s NCD division. They go to a higher office. Then, the project is brought to you as an order from above. So, in as much as on the technical level you are able to give these inputs and set conditions for when you are coming to do a NCD project, this is the biggest challenge.” (Interview #29, civil society representative)*

Misalignments between corporate and public health interests have produced huge inefficiencies in programming. For example, in one case a company prohibited an NGO from using funds provided to screen for diabetes to also screen for cardiovascular conditions, as the company was only interested in diabetes. However, government and civil society were often hesitant to push companies on adhering to partnership principles, as they fear the loss of corporate support.

*“So when they said, ‘no these are our priorities at the moment, ‘we had to make a decision and say, ‘Okay, it's better to focus on something than nothing.’ I think we just agreed to give in … It's just that we felt that maybe if we asked for too much they might decide to go elsewhere.” (Interview #44, civil society representative)*

For companies that are willing to ensure the alignment of their programs with national priorities, a broad set of national policies and disease-specific strategies exist and are easily accessible. However, on the county level priorities are less clear. While county development plans broadly address the health sector, few counties have laid out specific plans related to NCDs. Moreover, interviewees reported that it is difficult to get access to reliable data on disease prevalence and health systems capacities, making it difficult to understand the needs. Publicly provided data are incomplete in many cases; in other counties, the data simply do not exist:

*„The information system is also what we lack … we don't have a sufficient data registry on cancer that's national. So decisions are being made on emotional grounds.” (Interview #21, civil society representative).*

Still data availability is gradually improving, but some interviewees expressed concern that growing pressure to protect patient data more strictly might make it more difficult for non-state actors to access data in the future.

We need to point out that some companies voluntarily pushed for strong alignment of their programs with local priorities. One company for instance, invested time and resources into a bottom-up approach of developing a program improving cancer care. It invited stakeholders from different sectors for several meetings to discuss about priorities, intervention strategies and possible partners who now also lead and oversee the program’s implementation. In some cases, such companies were also able to build on ad-hoc support from the government to achieve alignment. Interviewees reported that the national Ministry of Health assigned technical staff to help develop program strategies, for example by advising on the selection of counties in which to operate:

*„We engaged with the Ministry of Health at high level who then cascaded it down and appointed a team to work with us.” (Interview #16, industry representative).*

The NCD division of the Ministry of Health has grown substantially; however, it still has limited human and financial resources when compared with other divisions in the Ministry. Thus, it could not assist the growing number of potential industry partners approaching it for support on an ad-hoc basis. In order to deal with limited resources, and to empower local stakeholders, the NCD division works closely with civil society organizations on governance of the sector. To this end, the NCD Strategy of 2015 proposed the creation of an NCD Interagency Coordinating Committee and various technical working groups (TWGs) on specific themes. Government representatives explained that companies are expected to submit their program ideas to the relevant TWG, thereby ensuring alignment and stakeholder involvement at the same time. However, the potential effectiveness of this structure has been undermined by budgetary constraints and frequent leadership changes in the Ministry of Health. While several TWGs have become operational, companies relied on their own networks for stakeholder engagement in previous years.

*“We selected the key players that we worked with at the time. Yet I was not sure if we got all the right and necessary stakeholders back then. Since then we did a lot of rejigging.” (Interview #31, industry representative)*

The Kenyan government has regularly called on companies to invest more in harmonization among their initiatives—this represents another informal norm. Many interviewees including government representatives complained publicly about inefficiencies that result from companies’ unwillingness to collaborate effectively.

*Everyone wants exclusivity, but I say: “The other company came last week and had almost the same program as you. Since you are not competing would you be able to partner so we use the same platform?”(Public statement, government representative* [45]*)*

Despite its calls, the government provides only limited support for harmonization. For example, there is no public registry of ongoing programs addressing NCDs. This makes it harder for potential new partners to obtain an overview of which actors are already working, in which counties, and on what issues. Government representatives also mentioned that initiatives are sometimes launched without their knowledge, especially when partners either go to the county level or decide to fund NGO activitiesdirectly.

*“I think that quite a number fall through the cracks and the Ministry also gets to know about them after private sector players have already engaged with the counties or facilities.” (Interview #24, government representative)*

In 2018, the national government and the international NGO PATH undertook a stakeholder mapping exercise that was supposed to be the foundation of a standing oversight platform. Several interviewees noted that this effort missed many initiatives and has not been regularly updated. These gaps are partially remedied by the existence of the Access Observatory, a database of programs financed by Access Accelerated [46]. However, the information listed in the Observatory is neither detailed enough for harmonization at the county level, nor does it include non-corporate NCD programs. Moreover, several interviewees suggested that the platform is not well-known among Kenyan stakeholders.

The government could improve harmonization by hosting regular joint planning forums with partners working on NCDs. We found a successful example of such a government-led multi-stakeholder NCD forum in the Kenyan cancer space – albeit without industry participation [34]. The TWGs of the NCD Interagency Coordinating Committee are providing such a platform in some disease areas, too. The Kenyan government has also hosted several broad NCD stakeholder meetings with the support of Access Accelerated; however, according to interviewees the conferences did not incorporate joint planning or coordination exercises and mainly had a representative function.

*“Understanding the dynamic and bringing people together, to have a conversation and to agree on the direction and on the implementation model—Access Accelerated currently has not done this very well. Everyone comes to the platform and showcases what they're doing differently and nothing is coordinated to exploit each other’s strengths and competences.”(Interview #1, industry representative)*

Beyond government, international organizations and major donors meet regularly at the Development Partners in Health roundtable for the purpose of harmonizing their programs. However, the roundtable has not yet put NCDs on its agenda. Further, it does not recognize corporations as development partners. Thus, companies cannot participate in its meetings. Moreover, we have found that - in some counties - existing multi-stakeholder consortia are assuming the role of ensuring harmonization and alignment of industry-led NCD initiatives. For example, the Academic Model for Providing Healthcare (AMPATH), a strategic partnership between Moi University, Moi Teaching and Referral Hospital and a group of North American and European Universities led by University of Indiana originally known for its HIV programs, has helped multiple pharmaceutical companies to develop and implement NCD initiatives in line with local needs, as well as harmonizing their corporate partners’ activities [30, 47]. Similarly, the “Blueprint for Innovative Healthcare Access” consortium of healthcare providers and NGOs tries to coordinate among multiple industry partners willing to launch NCD programs and points them to needs and gaps in the counties where it operates.

*“We look for partners to come in and help with the gaps that we are experiencing.” (Interview #34, civil society representative)*

### Partnership principles in program implementation

As summarized in Table 5, we found no laws or rules regulate how corporate programs should govern the programs they implement. Interviewees said that the public sector does not strongly promote monitoring and evaluation frameworks, nor does it place a high priority on their development. Thus, there are also no informal norms that could nudge companies towards adherence to the principle of managing by results.

*“The government did not give us clear requirements on reporting. There were some local standard things that local partners know and many were already collecting that information.” (Interview #31, industry representative)*

Programs that deliver health services, including screening, diagnosing or treating patients, are bound by local regulations to report on basic indicators - such as the number of cases of a specific disease treated - to the general Kenyan health information system. Yet, company representatives explained that the existing public health information system mostly captures output indicators. It is not sufficiently elaborate to inform a unified results framework that could guide outcome-oriented management for more complex NCD programs. To fill this gap, companies can draw on a repository of logic models and indicators offered by the Access Observatory, but some have resorted to independently developing their own results management frameworks. Moreover, in some cases, prominent implementing partners, for example the previously mentioned AMPATH consortium in Western Kenya, may push companies to install ambitious monitoring and evaluation systems in their NCD initiatives [47, 48].

**Table 5:** Kenya Results: Program governance

Partnership principles		Governance elements	Assessment questions	Assessment of the situation in Kenya
**Program governance**	Cross-cutting	Regulation	Do formal policies or legislation exist that regulate program governance?	• No rules exist addressing either internal accountability structures for industry-led programs or in how and how often they must report progress to the government. • Only programs that use health service delivery strategies, such as screening, diagnosing or treating patients, are bound by local regulation to report on basic output indicators to the Kenyan health information system.
			If not, do clearly stated informal norms exist of what government expects from companies in this regard?	• Weak norms exist. The public sector does not push strongly for M&E frameworks for programs and puts little priority on their development. • The only clear expectation is respect of government ownership, as shown by informing and inviting government representatives to events that relate to public sector responsibilities.
			In how far are these rules or norms backed up with sanctions to enforce compliance?	• There are informal sanctions in place.
	Managing by results	Results framework	Does the government provide a unified results framework that companies can build on?	• The existing country-wide health information system is not sufficient to guide the design of M&E frameworks. • Access Observatory (AO) has a repository of logic models and indicators for companies as a private alternative.
	Accountability	Reporting structures	Does the government provide a public reporting framework where results can be shared transparently?	• No official platform exists for Kenya. • AO allows companies to transparently report progress of their programs but it is not widely known or used in Kenya.
		Government oversight	Does government join governance structures of corporate programs?	• If invited, government representatives attend progress review meetings or sit on governance boards of programs. • However, government participation may be limited by staff capacities.
		Review meetings	Does government host regular review meetings where companies can report on progress?	• A few platforms exist for stakeholder exchanges. Some county-level governments organize regular stakeholder meetings. • The frequency and quality of county-level meetings vary. • Companies are rarely actively involved in such meetings. More often, companies are represented by their implementing partners. • Nationally, NCD-specific learning and exchange forums have taken place twice, through AA in cooperation with the MoH, but no permanent structure exists.

We identified very few norms, either formal or informal, regarding accountability. Government representatives stated in interviews that they wanted companies to respect their leadership and to inform them about and invite them to any activities related to the public sector, such as capacity development for public sector health workers or the dissemination of health management guidelines.

*“We do it in partnership with them. We would be very cross with them when they did the training without our involvement.” (Interview #24, government representative)*

Beyond that, however, there seemed to be no expectations regarding reporting modalities, transparency, or involving government and stakeholders in decision-making and oversight bodies. If invited by companies, government representatives agreed to join oversight or advisory bodies of programs with formal governance structures. However, government representatives explained that they do not have the capacity to be closely involved in individually monitoring all industry-led programs.

*“In terms of government monitoring, it is not an organized space. But it can only happen if there are functional technical working groups. Now there is the National Cancer Institute, but it is yet to be fully commissioned.” (Interview #21, civil society representative)*

Neither national nor county governments offer many supportive structures for companies proactively seeking to ensure accountability. No public reporting platform exists that companies could use to share their results and create transparency. Thus far, companies aiming to be publicly accountable use either the Access Observatory or their own websites for that purpose. However, most of the Kenyan stakeholders interviewed reported that they rarely used these channels to inform themselves about companies’ activities. Accountability could also be supported through regular progress review meetings with local stakeholders. However, these opportunities remain very limited. Some county governments do organize regular stakeholder meetings on health or even specifically on NCDs, during which implementing partners report on their activities and results. However, the frequency and quality of these forums varies widely from county to county.

*“In some counties, like in Kericho, the county calls meetings. Probably because of this one guy at a hospital who was really enthusiastic about the program. He made sure we had a quarterly meeting for a World Diabetes Foundation program. Very rigid quarterly meetings. They are important. Some counties do it, others don’t.” (Interview #23, civil society representative)*

These meetings are supposed to take place in each county on a regular basis as part of the general health governance system, but county governments generally depend on donor funding to support them. Moreover, the pharmaceutical companies are rarely actively involved, although they may be represented by their implementing partners. In some counties, interviewees were unaware of the corporate involvement in local NCD programs, as they only dealt with implementing partners that did not actively disclose their corporate funding.

*“When we started the program, the company was not part of it. All I knew is that of course this NGO has some heavy funding and maybe they were looking for counties where they could work. They never told me where their funding was coming from.” (Interview #40, county government representative)*

On the national level, NCD-specific learning and exchange forums involving corporations have taken place irregularly. In 2018 and 2019, national forums were hosted by the Ministry of Health in collaboration with the NCD Alliance Kenya, funded by Access Accelerated. Previous meetings also depended on donor funding preventing those meetings to function as an independent and regular accountability structure. With the outbreak of the COVID-19 pandemic, national NCD forums have been interrupted.

## Discussion

Our study examined how Kenya is approaching the challenge of governing industry-led NCD initiatives. We seek to contribute to an understanding of how countries can assure that industry-led public health initiatives adhere to partnership principles and become an effective development tool. Kenya provided an apt study setting given the large number of industry-led initiatives being implemented there.

We identified several governance features in use to steer industry-led programs in Kenya: Informal norms exist to guide companies that intend to launch a NCD program. Structures, such as TWGs and stakeholder forums, are in place at the national level and in some counties—these can make it easier for companies to adhere to partnership principles while taking the onus off them to organize to convene stakeholders. This indicates that Kenya has already made important strides to address the challenges related to involving the industry in its NCD response. Once finalized, the Kenya Health Sector Partnership and Coordination Framework currently in development will provide additional tools to further strengthen the governing of industry-led initiatives [41]. Another promising governance initiative that interviewees reported is underway is the government’s effort to strengthen the NCD Interagency Coordinating Committee by tasking it with the implementation and governance of an upcoming National Strategy for Prevention and Control of NCDs 2021–2025 which is yet to be published. Other LMICs aiming to work more closely with the pharmaceutical industry in their NCD response may find Kenya’s existing governance approaches informative.

However, many gaps still exist in Kenya’s approach to governance of industry-led health programming. At a minimum, these gaps could undermine the effectiveness of industry-led programs in fully contributing to the country’s NCD response. More problematically, they could have negative effects on the health system if poorly designed programs use up limited resources. The first gap is the lack of formal regulation or other official benchmarks to guide program strategies and governance. In the absence of formal guidance, companies can interpret partnership principles on a case-by-case basis and to their own advantage. Although the government has clear, if informal, expectations regarding alignment, harmonization, and ownership, no informal norms exist for accountability and results management. One possible explanation for this gap is a fear of government and civil society partners that regulation would deter companies from further investments in the NCD programs that are filling a need in Kenya. However, stakeholders may be underestimating the importance that the companies place on such programs, which form a key part of corporate non-market strategies by building up networks, improving corporate reputations, and creating an enabling environment for future commercial gains [49]. As a future growth market for the pharmaceutical industry [50], Kenya is highly relevant to many companies. Thus, we anticipate that companies would be likely to follow any regulations imposed—indeed, they might welcome more guidance on the government’s expectations. Interviewees pointed to positive experiences in some other countries, notably Rwanda, where the government has been more proactive in steering and setting conditions for industry involvement.

Second, while the public sector is trying to expand enabling governance practices, many are not yet functioning well. The existing ad-hoc support for program design varies widely. Currently, the government, both nationally and in many counties, has not allocated resources to uphold and sufficiently finance permanent and robust structures that companies and other partners could utilize for better adherence to partnership principles. For example, stakeholder forums are supposed to be held regularly on the county level according to health governance regulations, but rarely take place. Instead, most enabling governance structures are currently provided by non-governmental actors, such as the accountability function of the Access Observatory [46]. Still, privately supported governance function could also only partially steer industry-led NCD initiatives. While important implementing partners such as AMPATH that work with multiple companies have leverage and expertise to shape strategy and implementation of corporate programs, these are only present in some counties or disease areas. National-level structures such as Access Accelerated and the Development Partners in Health roundtable are missing opportunities to promote harmonization. It is notable that while the traditional donors for health are not significantly supporting NCD interventions, they are also not coordinating or harmonizing with the industry players that are filling that gap.

Third, we want to specifically highlight the limited availability of epidemiological and health service data as a cross-cutting factor undermining coordination among stakeholders—this is a challenge that extends beyond just governing industry-led programs. Building up data repositories has been a factor for success in other areas of global health. For example, national governments have closely coordinated with external agencies to expand data collection and use in, for example, both HIV [51] and malnutrition [52]. Expanding collection of and access to data will also be a key element for better governance of the NCD sphere.

Many interviewees recognized the existing gaps in regulating and enabling governance and expressed support for closing them. Kenya has a window of opportunity to use this current stakeholder support to develop and implement stronger governance of industry-led public health programs. Traditional development donors could play a more active role by supporting Kenya and other countries on this path, for instance by contributing to ongoing efforts around the UN Sustainable Development Goals (SDG) Partnership Platform [53]. For such efforts to succeed, companies must be willing to cooperate and stop circumventing established processes, for example by using ties to high-level political leaders who make exceptions for them [54].

In addition to making recommendations specific to the Kenyan NCD context, this paper presents a tool for assessing national governance of other industry-led public health programs. While many scholars have argued for better governance of private sector involvement in public health, these calls lack specificity about what they imply for countries [22, 55]. Similarly, previous work analyzing country-level implementation of the Paris Declaration has ignored the roles of corporations as development partners [56], while focusing on specific aspects such as donor coordination [57]. While we developed the assessment tool for the case of pharmaceutical industry-led NCD programs, it could easily be adapted for use either in other sectors in public health or with other industries.

### Limitations

Our assessment tool sought to integrate principles of aid effectiveness with perspectives on governance of corporate initiatives in a new way. Therefore, we could not adopt an existing validated framework. We do not claim that the proposed framework and tool are complete. Instead, we propose this tool as a starting point for further use, discussion, and development. For example, we focused only on how industry-led programs can be governed for effectiveness. Governance processes might also address other concerns, such as how to decide the degree to which a national health system is willing to work with industry-led programs in the first place.

The application of our assessment tool to the Kenyan NCD context was limited by the available data. As the local governance system is dynamic, official documents were often outdated or incomplete. Thus, we had to draw on interview accounts to expand the picture. While we tried to triangulate insights across interviews and by feeding preliminary results back to experts, we might have missed some relevant information.

## Conclusion

This paper presented an assessment tool to study how a country governs pharmaceutical industry-led public health programs, which are growing in number—especially in LMICs. By using the tool to examine the case of the Kenyan NCD response, we generated insight into how normative calls for stronger country-level governance are being implemented. Other countries with growing pharmaceutical companies’ involvement in their NCD response may learn from Kenya about formulating clear rules of engagement and creating a supportive environment. For Kenya, these findings could contribute to the forthcoming health partnership framework and implementation of the new strategy for prevention and control of NCDs2021–2025.

The study highlights that LMIC governments face multiple challenges in developing and implementing comprehensive and functioning governance systems that can regulate and steer industry-led programs in public health. If traditional donors continue to provide only limited support to address NCDs, and as corporations become more active in working towards the SDGs, countries require support to strengthen the regulatory and enabling structures that can assure that industry-led programs become an effective development tool.

## Data Availability

The datasets generated and/or analyzed during the current study are not publicly available to ensure privacy of interviewees. Access to anonymized interview transcripts and archival data is available from the corresponding author on reasonable request.
